# Effects of HA1, a probucol analogue, and APOC3 siRNA on lipoprotein and amyloid-β metabolism and neurovascular dysfunction in type 2 diabetic mice

**DOI:** 10.3389/fphar.2026.1826957

**Published:** 2026-09-01

**Authors:** Arazu Sharif, John Charles Louis Mamo, Virginie Lam, Giuseppe Luna, Gerald F. Watts, Michael Leo Nesbit, Hani Al-Salami, Armin Mooranian, Ryusuke Takechi

**Affiliations:** 1 Curtin Medical Research Institute, Faculty of Health Sciences, Curtin University, Perth, WA, Australia; 2 Perron Institute for Neurological and Translational Research, Perth, WA, Australia; 3 Curtin Medical School, Faculty of Health Sciences, Curtin University, Perth, WA, Australia; 4 School of Public Health, Faculty of Health Sciences, Curtin University, Perth, WA, Australia; 5 School of Medicine, University of Western Australia, Perth, WA, Australia; 6 Department of Cardiology, Royal Perth Hospital, Perth, WA, Australia

**Keywords:** amyloid-β, anxiety-like behaviour, APOC3 siRNA, blood–brain barrier, lipoprotein, neurovascular dysfunction, probucol analogue, type 2 diabetes

## Abstract

**Introduction:**

Chronic vascular exposure to elevated levels of lipoprotein-amyloid-β (Aβ) contributes to blood–brain barrier (BBB) disruption and the pathogenesis of Alzheimer’s disease (AD). Therapeutic agents such as probucol have been shown to reduce circulating lipoprotein-Aβ levels and mitigate neurovascular dysfunction and cognitive decline. Type 2 diabetes (T2D) impairs BBB integrity and is characterised by dyslipidaemia and metabolic disturbances that may influence the peripheral metabolism of lipoprotein-Aβ. However, the effects of diabetes on lipoprotein-Aβ homeostasis and neurovascular integrity remain poorly understood. This study investigated whether diabetes impairs lipoprotein and Aβ metabolism and whether treatment with HA1, a probucol analogue with improved bioavailability, or APOC3 siRNA can modify these metabolic changes and improve neurovascular and behavioural outcomes.

**Methods:**

Non-diabetic db/+ mice, untreated diabetic db/db mice, and diabetic db/db mice treated with HA1 or APOC3 siRNA were used to determine how diabetes and therapeutic modulation of lipoprotein metabolism affect circulating Aβ, neurovascular integrity, and behavioural outcomes. Total plasma Aβ and ApoB levels were measured by ELISA. Intestinal Aβ and ApoB, neurovascular integrity, neuroinflammation, and oxidative stress were assessed by immunofluorescence. Anxiety-like behaviour and short- and long-term memory were evaluated using the open field, novel object recognition, and passive avoidance tests

**Results:**

Diabetic db/db mice exhibited increased intestinal Aβ and ApoB immunoreactivity and elevated total plasma Aβ_42_, Aβ oligomer and ApoB, accompanied by heightened BBB permeability and anxiety-like phenotype. HA1 reduced intestinal Aβ and lowered plasma Aβ_42_ and Aβ oligomer, prevented IgG extravasation, and improved anxiety. APOC3 siRNA lowered plasma ApoB and plasma Aβ_42_ and attenuated neuroinflammation, but did not reduce IgG extravasation.

**Conclusion:**

Dysregulated lipoprotein and Aβ metabolism are associated with neurovascular dysfunction and anxiety-like behaviour in diabetes. HA1 shows therapeutic potential by modulating Aβ metabolism and improving neurovascular and behavioural outcomes.

## 1 Introduction

Diabetes mellitus, particularly type 2 diabetes (T2D), is associated with cognitive impairment, anxiety, and an increased risk of dementia, including Alzheimer’s disease (AD) and related dementias (Gudala et al., 2013; Smith et al., 2013). Indeed, more than 80% of individuals with AD have been reported to exhibit T2D or abnormal glucose metabolism. Consistent with this, a growing body of evidence suggests that the pathogenesis of diabetes-associated cognitive decline and AD involves the interaction of multiple pathological processes, including metabolic dysregulation, vascular dysfunction, chronic inflammation, and oxidative stress. Among these, blood–brain barrier (BBB) dysfunction is recognised as an early and key contributor to neuronal injury and cognitive decline. However, the mechanisms linking systemic metabolic disturbances to neurovascular dysfunction remain incompletely understood.

Peripheral lipogenic organs, including the liver and small intestine, have been shown to synthesise amyloid-β (Aβ) and secrete it into the circulation in association with lipoproteins (Galloway et al., 2007; Galloway et al., 2009; Koudinov and Koudinova, 1997). Matsubara et al. reported that approximately 90% of circulating Aβ1–40 and 97% of Aβ1–42 are lipoprotein-bound (Matsubara et al., 2004), particularly within triglyceride-rich lipoprotein (TRL) fractions. Kinetic studies further indicate that Aβ remains associated with the lipoprotein particle and follows its catabolic pathway until hepatic clearance (James et al., 2003). Growing evidence suggests that chronic vascular exposure to elevated levels of lipoprotein- Aβ contributes to neurovascular pathology in AD (Takechi et al., 2023) (Takechi et al., 2023; Nakamura et al., 2018). Preclinical models have shown that diets high in fat can exacerbate the secretion of TRL-Aβ, leading to BBB disruption and the extravasation of this complex into the brain parenchyma (Galloway et al., 2007; Galloway et al., 2019; Takechi et al., 2010a; Takechi et al., 2009). Once within the brain parenchyma, these complexes trigger neurovascular inflammation, neuronal loss and cognitive deficits (Takechi et al., 2023; Takechi et al., 2010a). Particularly compelling evidence has come from genetically engineered mice that synthesise human Aβ exclusively in the liver, showing a clear peripheral contribution of Aβ to neurodegeneration (Lam et al., 2021).

In the context of diabetes, insulin resistance may promote TRL-Aβ secretion, potentially exacerbated by metabolic disruptions such as hyperphagia, hypertrophy, obesity and dysregulated hepatic lipid metabolism. This “endocrine axis” of TRL-Aβ secretion in insulin resistance may increase peripheral Aβ burden and, in turn, contribute to BBB vulnerability. Previous studies in mouse models investigating high-fat diet-induced diabetes are consistent with a lipoprotein-amyloid and capillary axis for disease but confounded by the independent effects of dietary fat on the metabolic and neurovascular systems (Takechi et al., 2010b; Takechi et al., 2008; Burgess et al., 2006). These models do not fully capture the impact of insulin resistance (Lin et al., 2016)—an underlying feature of type 2 diabetes that plays a critical role in both systemic and central metabolic dysfunction. Studies using rodent models of insulin resistance-induced diabetes (without high-fat diet interventions) would provide a clearer picture of how this lipoprotein-Aβ/capillary axis drives BBB breakdown, neuroinflammation, and cognitive decline.

Impaired hepatic clearance of TRL-Aβ and prolonged vascular exposure we postulate would allow these complexes to accumulate at the BBB, where they compromise barrier function. Considering these putative physiological aberrations in peripheral metabolism of lipoprotein-Aβ may offer a novel therapeutic approach to mitigating BBB dysfunction and cognitive decline in diabetic patients.

Probucol, a lipid-lowering agent with antioxidant and anti-inflammatory properties, has shown promise in reducing circulating TRL-Aβ levels and protecting BBB integrity in models of metabolic dysfunction, including high-fat diet-induced diabetes (Sharif et al., 2024; Takechi et al., 2013; Takechi et al., 2014). However, its clinical utility is limited by its poor bioavailability (Mamo et al., 2018). HA1, a novel probucol analogue conjugated with lithocholic acid (LCA), has demonstrated improved pharmacokinetics and may have greater therapeutic potential. In parallel, apolipoprotein C-III (ApoC3) is a key regulator of TRL metabolism that delays lipoprotein clearance by inhibiting lipolysis and hepatic uptake. Targeting *Apoc3* using small interfering RNA (siRNA) therefore represents an alternative strategy to reduce circulating TRLs and associated circulating Aβ.

In this study, we aimed to explore the role of lipoprotein and Aβ in insulin resistance-induced BBB dysfunction and neurovascular inflammation using leptin receptor-deficient db/db mice. We further investigated whether interventions with HA1 or *APOC3* siRNA could modify TRL and Aβ measures, protect BBB integrity and prevent neuroinflammation, anxiety and cognitive decline.

## 2 Materials and methods

### 2.1 Animals

All procedures were approved by the Curtin Animal Ethics Committee (ARE 2020-12) and complied with the Australian Code for the Care and Use of Animals, 8th edition. Five-week-old male db/db and db/+ C57BL/6J mice were obtained from The Jackson Laboratory and bred at the Animal Resource Centre (WA). Mice were housed with controlled air pressure, 12:12 light/dark cycles, and temperature at Curtin University’s Life Science Research Facility.

After 1 week of acclimation, db/+ mice were maintained on standard chow (AIN93M; Specialty Feeds, WA) and served as non-diabetic controls. Diabetic db/db mice were randomly assigned (n = 12–14 per group) to one of the following groups: standard chow alone, standard chow supplemented with 1.8% HA1 (2,6-di-tert-butyl-4-((2-((3,5-di-tert-butyl-4-hydroxyphenyl)thio)propan-2-yl)thio)phenyl (4R)-4-[(3R,5R,8R,9S,10S,13R,14S,17R)-3-acetoxy-10,13-dimethyl-2,3,4,5,6,7,8,9,11,12,14,15,16,17-tetradecahydro-1H-cyclopenta [a]phenanthren-17-yl]pentanoate), or standard chow with four weekly subcutaneous injections of APOC3 siRNA (5 mg/kg). APOC3 siRNA was generously provided by Arrowhead Pharmaceuticals, Inc.

All mice had *ad libitum* access to food and water throughout the study.

### 2.2 Measurement of anxiety and cognitive function

The open field test (OFT) was used to assess anxiety-like behaviour. At 28 weeks of age, mice were acclimated to a dimly lit testing room for 20 min and then placed individually in a square arena (45 × 45 cm, 40 cm high) where they were allowed to explore freely for 5 min. The time spent in the periphery of the arena was recorded as an index of anxiety-like behaviour.

Following the OFT, novel object recognition (NOR) and passive avoidance (PA) tests were used to assess short- and long-term memory, respectively. In the NOR test, mice were first familiarised with two identical objects and, after a 2-h retention interval, tested for preference for a novel object to calculate the Preference Index. In the PA test, mice were trained in a two-compartment apparatus in which entry into the dark compartment was paired with a foot shock. Memory retention was assessed 24 h later based on latency to enter the dark compartment.

### 2.3 Sample collection

Mice were euthanised by cardiac puncture under isoflurane anaesthesia. All samples were collected under *ad libitum* (non-fasted) conditions, with free access to chow and water maintained until the time of sampling. Blood was collected into EDTA-coated tubes, and plasma was isolated by centrifugation at 3,000 rpm for 10 min at 4 °C and stored at −80 °C. A 2 cm segment of proximal duodenum was excised and fixed in 4% paraformaldehyde, then processed and embedded in paraffin wax. The brain was carefully removed; the left hemisphere was snap-frozen in liquid nitrogen, while the right hemisphere was fixed in 4% paraformaldehyde for 24 h, cryoprotected in 20% sucrose for 72 h at 4 °C, frozen in isopentane, and stored at −80 °C.

### 2.4 Measurement of plasma glucose and insulin

Plasma glucose concentrations were measured using a colorimetric assay kit (Cayman Chemical), and plasma insulin concentrations were quantified using a Mouse Insulin ELISA kit (Mercodia), according to the manufacturers’ instructions. Optical density was measured using an EnSight Multimode Plate Reader at 500 nm for glucose and 450 nm for insulin.

### 2.5 Measurement of plasma cholesterol, triglycerides and ApoB

Plasma cholesterol and triglyceride concentrations were measured using colorimetric assay kits (Randox Laboratories, United Kingdom) according to the manufacturer’s protocol. Briefly, 2 µL of standards or plasma samples were mixed with 200 µL of reaction solution in 96-well plates and incubated at 37 °C for 5 min. Absorbance was measured at 550 nm, and lipid concentrations were calculated from standard curves.

Plasma ApoB concentrations were quantified using an ELISA kit (Abcam) according to the manufacturer’s instructions. Plasma samples from db/+ mice were diluted 1:3000, whereas samples from db/db and treated db/db mice were diluted 1:6000 in the provided dilution buffer. Optical density was measured at 450 nm, 15 min after addition of the stop solution.

### 2.6 Immunofluorescent detection of intestinal Aβ and ApoB

Intestinal Aβ and ApoB expression were quantified by immunofluorescence microscopy as previously described (Galloway et al., 2019; Pallebage-Gamarallage et al., 2012). A 2 cm segment of proximal duodenum from each animal was fixed, paraffin-embedded, sectioned at 5 μm, deparaffinised, and rehydrated, followed by antigen retrieval in boiling deionised water for 15 min. Sections were permeabilised with phosphate-buffered saline (PBS) containing Tween-20 for 10 min and blocked with 20% goat serum for 1 h at 20 °C.

Sections were then incubated overnight at 4 °C with rabbit anti-mouse Aβ(1–40/42) antiserum (1:200; Millipore) or polyclonal rabbit anti-mouse ApoB antibody (1:200; Abcam) diluted in blocking solution. Immunofluorescence was visualised using Alexa Fluor 546–conjugated anti-rabbit IgG (1:100; Thermo Fisher Scientific), and nuclei were counterstained with Hoechst 33342 (1:2000; Thermo Fisher Scientific).

Duodenal sections were scanned using a Zeiss Axioscan Z.1 slide scanner (Carl Zeiss, Germany) with identical acquisition settings within each staining run. Prior to quantification, images were reviewed to confirm staining consistency and absence of signal saturation within the analysed regions. For each animal, Aβ and ApoB fluorescence intensity were quantified from raw images across one full duodenal section, with analysis restricted to the absorptive villus region. A uniform fluorescence threshold was applied across all sections within each staining run. Regions affected by tissue damage, folds, edge effects, staining artefacts, or imaging artefacts were excluded from analysis.

Total Aβ or ApoB fluorescence intensity was normalised to total nuclear area within the analysed region to account for differences in cell density and section area between samples. Duodenal sections from each animal were analysed individually, and final data are expressed as mean ± SEM of Aβ or ApoB fluorescence intensity per total nuclear area.

### 2.7 Plasma Aβ analysis

Plasma Aβ40, Aβ42, and Aβ oligomer levels were measured separately using commercially available analyte-specific ELISA kits (Wako; catalogue numbers 294-64701, 292-64501, and 290-82001, respectively), according to the manufacturer’s protocol. Concentrations were determined from standard curves generated using Aβ_40_ standards ranging from 0 to 100 pmol/L, Aβ_42_ standards ranging from 0 to 20 pmol/L, and Aβ oligomer standards ranging from 0 to 100 pmol/L.

### 2.8 Assessment of BBB integrity and tight junction protein and pericyte expression

BBB integrity was evaluated by quantifying extravasated plasma IgG in the cortex and hippocampus as previously described (Nesbit et al., 2021). Coronal brain sections (20 µm) from snap-frozen left hemispheres were fixed in 4% paraformaldehyde (PFA) for 10 min, blocked with 10% donkey serum for 1 h at 20 °C, and incubated overnight at 4 °C with goat anti-laminin α4 (1:200; R&D Systems) diluted in ASEB buffer. Sections were then incubated with donkey anti-goat Alexa Fluor 555 (1:500; Abcam) for 2 h at 20 °C, followed by Alexa Fluor 488–conjugated secondary antibodies for detection of endogenous IgG. Nuclei were counterstained with Hoechst 33342 (1:500; Thermo Fisher Scientific).

For immunofluorescent detection of tight junction proteins, 20 µm brain sections were fixed in 4% PFA followed by permeabilisation with ice-cold acetone/methanol (50:50). Sections were blocked with 10% donkey serum and incubated overnight at 4 °C with goat anti-laminin α4 (1:200; R&D Systems) together with rabbit anti-occludin (1:500; Thermo Fisher Scientific) or rabbit anti–zonula occludens-1 (ZO-1; 1:500; Thermo Fisher Scientific). Pericyte coverage was assessed using a similar protocol with rabbit anti-PDGFRβ (1:100; Cell Signaling Technology). Appropriate Alexa Fluor–conjugated secondary antibodies were applied for 2 h at 20 °C.

Fluorescent images were acquired at ×20 magnification using a Zeiss Axioscan Z.1 slide scanner under identical acquisition settings within each staining run. Quantification was performed on raw images using Zeiss ZEN software. IgG leakage was quantified using the Zeiss ZEN Intellesis trainable segmentation module, as previously described (Nesbit et al., 2021). Briefly, the segmentation model was trained on representative images to distinguish vascular structures from surrounding parenchyma and was then applied uniformly across all images within each staining run. IgG leakage was quantified as extravascular IgG fluorescence signal within the parenchymal compartment. PDGFRβ, occludin, and ZO-1 fluorescence intensity were quantified within segmented vascular regions using the same software platform. For all vascular markers, voxel intensity sum was normalised to vascular area.

For each animal, one section per staining run was analysed. The cortex and hippocampus were identified using standard anatomical landmarks, with reference to the Allen Mouse Brain Atlas (Wang et al., 2020). The cortex and hippocampus were analysed by dividing each region into fixed-size, non-overlapping ROIs using ZEN software, with a minimum of 20 ROIs analysed per brain region per animal. Regions affected by tissue damage, folds, edge effects, staining artefacts, or imaging artefacts were excluded. ROI-level values were averaged to generate a single animal-level value for each brain region for statistical analysis. Image acquisition and ROI placement were not performed blinded to treatment group.

### 2.9 Measurement of neuroinflammation, oxidative stress, and axonal and neuronal density

Neuroinflammation and oxidative stress were assessed in both the cortex and hippocampus, while neuronal and axonal density were quantified in the hippocampus, as previously described (Lam et al., 2021). Coronal sections (20 µm) from the right hemisphere were rehydrated in phosphate-buffered saline (PBS), blocked with 10% donkey serum for 1 h at room temperature, and incubated overnight at 4 °C with the following primary antibodies diluted in ASEB buffer: goat anti–glial fibrillary acidic protein (GFAP; 1:500; Abcam) for astrocytes, rabbit anti–ionised calcium-binding adaptor molecule 1 (Iba-1; 1:200; Novachem) for microglia, mouse anti–8-hydroxy-2′-deoxyguanosine (8-OHdG; 1:500; Abcam) for oxidative DNA damage, mouse anti-SMI312 (1:500; BioLegend) for axonal density, and mouse anti-NeuN (1:500; Merck) for neuronal density.

Sections were incubated with appropriate secondary antibodies, including donkey anti-rabbit Alexa Fluor 488 (1:500; Thermo Fisher Scientific), donkey anti-goat Alexa Fluor 555 (1:1000; Thermo Fisher Scientific), and donkey anti-mouse Alexa Fluor 647 (1:500; Thermo Fisher Scientific). Nuclei were counterstained with Hoechst 33,342 (1:500; Thermo Fisher Scientific).

Fluorescent images were acquired at ×20 magnification using a Zeiss Axioscan Z.1 slide scanner (Carl Zeiss, Germany) under identical acquisition settings within each staining run and quantified on raw images using the Zeiss ZEN Intellesis trainable segmentation module. The model was trained on representative images to identify positively stained GFAP-, Iba-1, and 8-OHdG-positive cells and applied uniformly across all images. GFAP and Iba-1 staining intensity was quantified per image, while 8-OHdG fluorescence intensity was normalised to nuclei count within the same analysed regions.

ROI definition, artefact-exclusion criteria, and animal-level averaging were performed as described in Section 2.8. Briefly, a minimum of 20 fixed-size, non-overlapping ROIs were analysed per brain region per animal, and ROI-level values were averaged to generate a single animal-level value for each brain region. For SMI312 and NeuN analyses, the dentate gyrus was delineated manually using anatomical landmarks, and fluorescence intensity was quantified within the defined region. Intensity sum for each marker was normalised to the analysed image area.

### 2.10 Statistical analysis

All data are presented as mean ± SEM. Data were assessed for normality prior to statistical analysis. Normally distributed data were analysed using one-way ANOVA followed by Fisher’s LSD *post hoc* test. Comparisons were restricted to db/db versus db/+ mice to assess diabetes-associated differences, and db/db mice versus HA1-or APOC3 siRNA-treated db/db mice to assess treatment effects. Non-normally distributed data were analysed using the Kruskal–Wallis test followed by uncorrected Dunn’s test for the same planned comparisons, with the alpha threshold set at 0.05. Associations between variables were assessed using Spearman’s rank correlation coefficient, with 95% confidence intervals reported. Correlation analyses were performed using continuous quantitative variables and were intended to assess exploratory associations between measures derived from different experimental domains. Statistical significance was defined as p < 0.05, with p < 0.01 and p < 0.001 reported where applicable.

## 3 Results

### 3.1 Effects of HA1 and APOC3 siRNA on metabolic parameters

**FIGURE 1:**
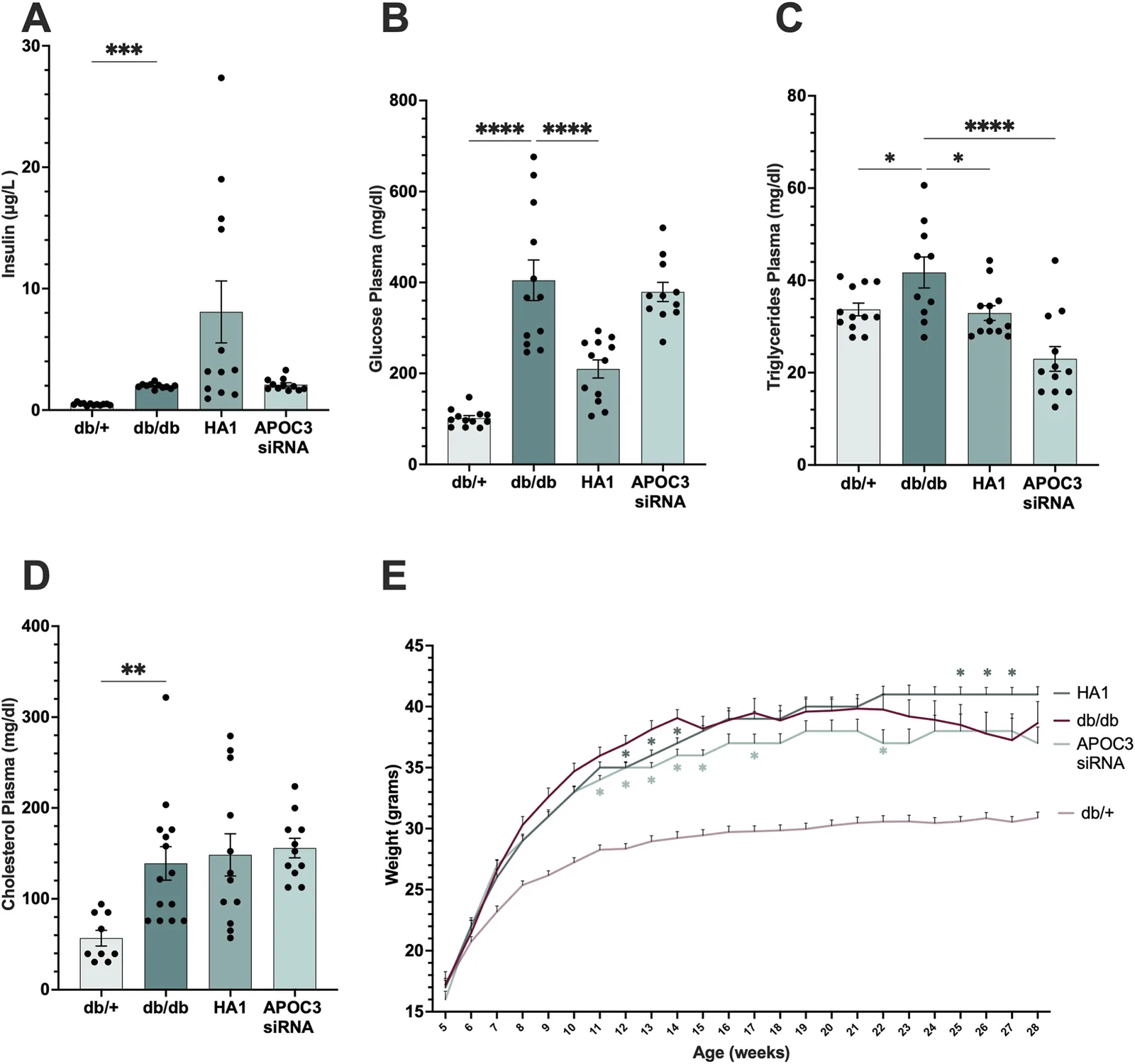
Metabolic parameters in db/db and db/+ mice. Non-fasting plasma insulin **(A)**, non-fasting plasma glucose **(B)**, plasma triglycerides **(C)**, plasma cholesterol **(D)**, and body weight **(E)**. Data are presented as mean ± SEM. Statistical analyses were performed using one-way ANOVA followed by Fisher’s LSD *post hoc* test for glucose, triglycerides, and body weight, and the Kruskal–Wallis test followed by uncorrected Dunn’s multiple-comparison test for insulin and cholesterol (*p < 0.05, **p < 0.01, ***p < 0.001, ****p < 0.0001).

Diabetic db/db mice exhibited significantly elevated plasma insulin, glucose, triglycerides, and cholesterol compared with age-matched db/+ controls (Figures 1A–D). HA1 treatment significantly reduced plasma glucose and triglyceride levels and was associated with a non-significant increase in plasma insulin. APOC3 siRNA treatment did not significantly alter plasma glucose or insulin concentrations but markedly reduced plasma triglyceride levels. Neither HA1 nor APOC3 siRNA affected plasma cholesterol levels.

Both HA1- and APOC3 siRNA–treated db/db mice exhibited reduced body weight gain compared with untreated db/db controls, with significant differences observed up to 14 and 23 weeks of age, respectively (Figure 1E). In addition, both treatments attenuated the progressive weight loss typically observed in diabetic db/db mice at later stages.

### 3.2 HA1 lowered the increase in intestinal Aβ expression in db/db mice

**FIGURE 2:**
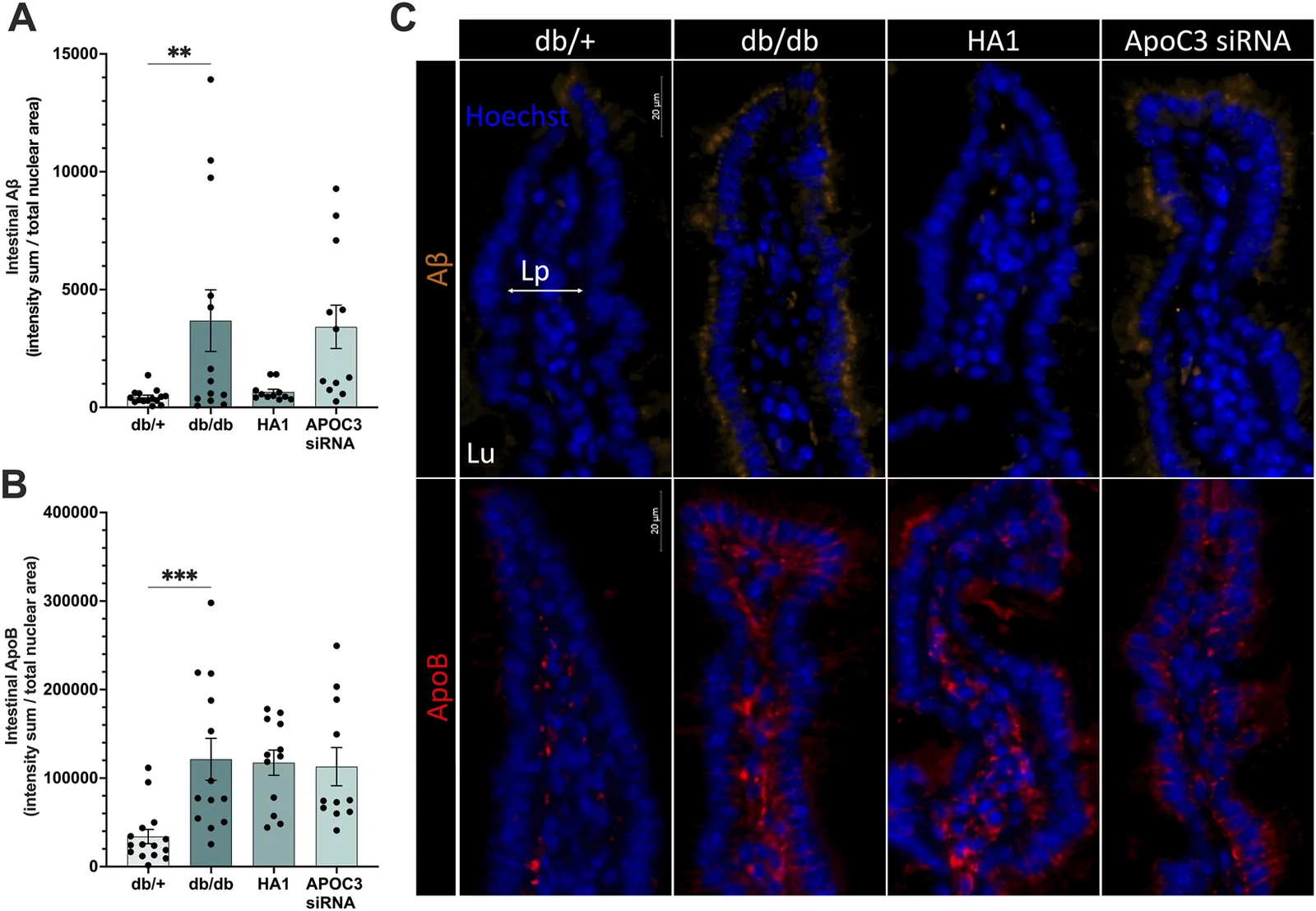
Intestinal abundance of Aβ and ApoB. Quantification of intestinal Aβ fluorescence intensity normalised to nuclear area **(A)** and intestinal ApoB fluorescence intensity normalised to nuclear area **(B)**. Representative immunofluorescent micrographs showing Aβ (orange) and ApoB (red) in intestinal sections **(C)**. The white arrow indicates lamina propria (Lp) and Lu labels lumen. Scale bar = 20 µm. Data are presented as mean ± SEM. Statistical significance was determined using the Kruskal–Wallis test followed by uncorrected Dunn’s multiple-comparison test (n > 12, *p < 0.05, **p < 0.01, ***p < 0.001, ****p < 0.0001).

Aβ and apolipoprotein B (ApoB)—an obligatory structural component of nascent chylomicrons—were quantified within the absorptive villus region as a surrogate measure of intestinal amyloid and nascent lipoprotein abundance. Diabetic db/db mice exhibited a significant increase in intestinal Aβ immunoreactivity compared with db/+ controls (Figures 2A,C). Treatment with HA1 markedly reduced intestinal Aβ abundance in db/db mice, whereas APOC3 siRNA had no significant effect (Figures 2A,C).

In parallel, intestinal ApoB immunoreactivity was significantly increased compared with db/+ controls (Figures 2B,C), consistent with altered intestinal lipoprotein metabolism in db/db mice. Neither HA1 nor APOC3 siRNA significantly altered intestinal ApoB levels.

### 3.3 HA1 and APOC3 siRNA reduced plasma Aβ_42_ and oligomeric Aβ levels

**FIGURE 3:**
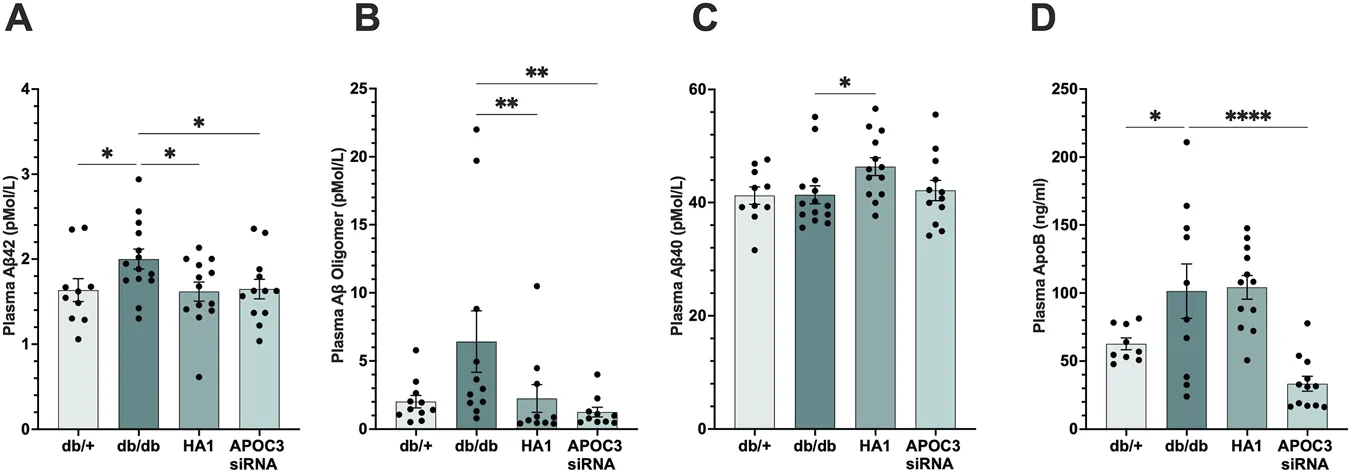
Concentrations of plasma Aβ and ApoB. Total plasma Aβ_42_ **(A)**, Aβ oligomers **(B)**, Aβ_40_ **(C)** and plasma ApoB **(D)** are shown. Data are presented as mean ± SEM (n = 11–15 mice per group). Statistical significance was determined using one-way ANOVA for Aβ_42_ and ApoB, and the Kruskal–Wallis test followed by uncorrected Dunn’s multiple-comparison test for Aβ_40_ and Aβ oligomers (*p < 0.05, **p < 0.01, ***p < 0.001).

The more aggregation-prone Aβ isoform, Aβ_42_, was significantly elevated in the plasma of db/db mice compared with db/+ controls (Figure 3A). In addition, plasma Aβ oligomers, often used as surrogate markers of increased amyloid aggregation propensity, were increased by approximately 40% in db/db mice at 28 weeks of age (Figure 3B). Treatment with either HA1 or APOC3 siRNA significantly reduced total plasma Aβ_42_ and Aβ oligomer levels.

Notably, the patterns of reduction differed between interventions. APOC3 siRNA markedly reduced plasma ApoB levels, consistent with decreased circulating ApoB-containing lipoproteins that carry a substantial proportion of plasma Aβ (Figure 3D). In contrast, HA1 treatment did not significantly alter plasma ApoB levels (Figure 3D), suggesting that its effects on plasma Aβ occur independently of changes in circulating lipoprotein abundance.

In contrast to Aβ_42_, plasma Aβ_40_ levels were not significantly altered by diabetes (Figure 3C). HA1 treatment was associated with a modest increase in plasma Aβ_40_, whereas APOC3 siRNA had no significant effect on Aβ_40_ levels.

### 3.4 Intestinal levels of ApoB and Aβ correlates with plasma ApoB and Aβ42

**FIGURE 4:**
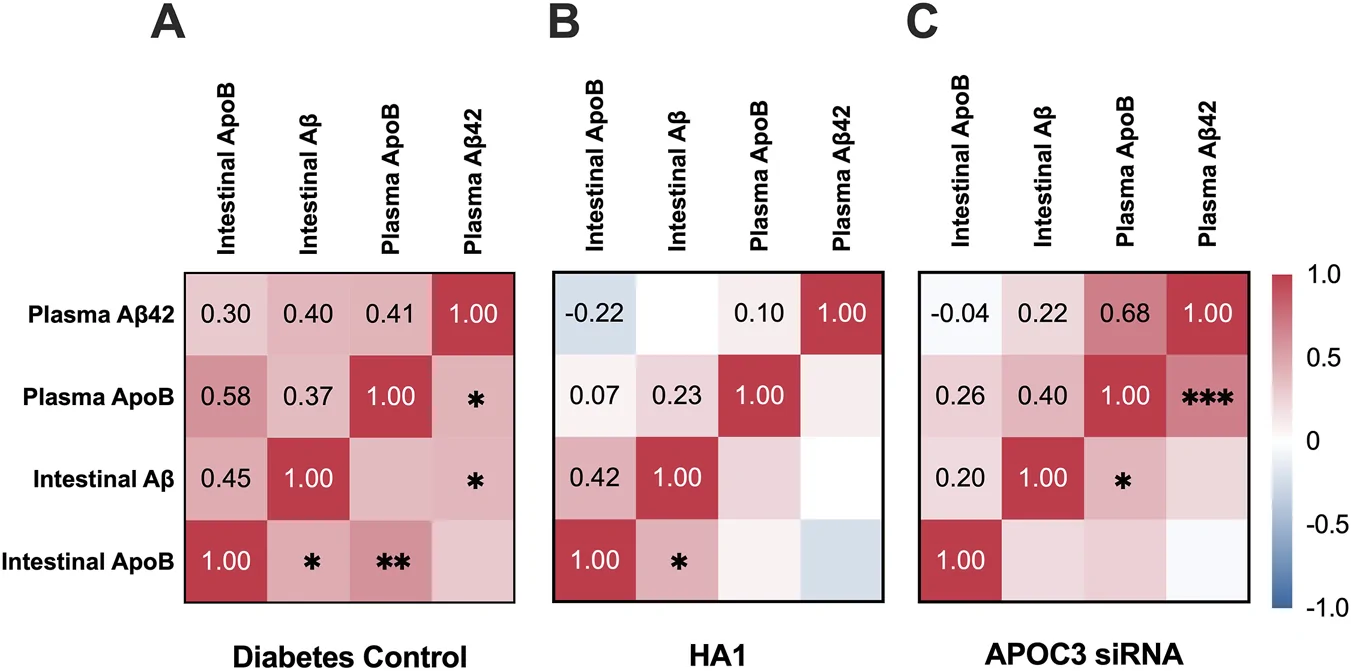
Correlation analysis of intestinal and plasma amyloid measures. Spearman’s rank correlation analyses examining relationships between intestinal Aβ, intestinal ApoB, plasma Aβ_42_, and plasma ApoB in combined db/+ and untreated db/db control mice **(A)**, db/db mice treated with HA1 **(B)**, and db/db mice treated with APOC3 siRNA **(C)**. Spearman’s correlation coefficients (r) with 95% confidence intervals are shown in the upper triangle, and corresponding statistical significance values are shown in the lower triangle (*p < 0.05, **p < 0.01, ***p < 0.001; n = 21–26).

Figure 4 examines the relationship between intestinal Aβ and ApoB immunoreactivity vs. plasma Aβ_42_ and ApoB levels. In control db/db and db/+ mice (Figure 4A), intestinal Aβ was strongly associated with intestinal ApoB, and each marker correlated positively with its corresponding plasma measure. These correlations support the hypothesis that intestinal amyloid and lipoproteins may contribute to circulating Aβ_42_ and lipoprotein levels.

In db/db mice treated with HA1 (Figure 4B), the association between intestinal Aβ and intestinal ApoB was maintained, whereas the association between intestinal Aβ and plasma Aβ_42_ was no longer significant. The association between plasma Aβ_42_ and plasma ApoB was also attenuated. Together, these findings suggest that HA1 decouples intestinal Aβ abundance from circulating Aβ_42_, supporting the hypothesis that HA1 lowers plasma Aβ_42_ partly by reducing the intestinal contribution to circulating Aβ.

In db/db mice treated with APOC3 siRNA (Figure 4C), the association between intestinal Aβ and intestinal ApoB was reduced. In contrast, the preserved association between plasma Aβ_42_ and plasma ApoB, together with the reduction in both circulating markers, suggests that APOC3 siRNA may lower plasma Aβ_42_ in association with ApoB-containing lipoproteins.

### 3.5 HA1 increases ZO-1 and pericyte expression, and mitigates BBB dysfunction

**FIGURE 5:**
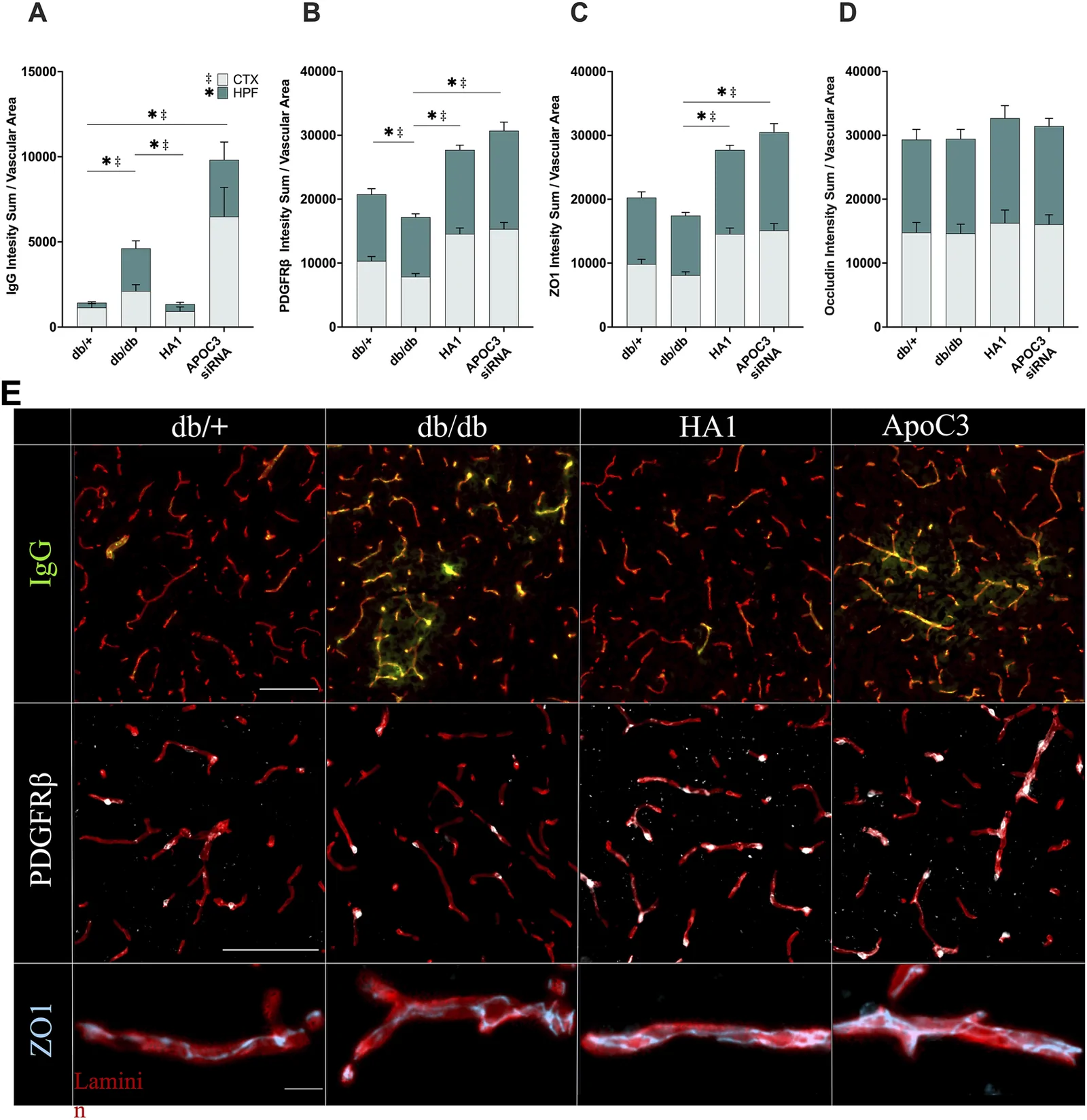
Immunomicroscopic analysis of cerebral capillary integrity, pericyte coverage, and tight junction protein expression. Hippocampal (HPF) and cortical (CTX) extravasation of plasma IgG, expressed as IgG intensity sum per laminin area **(A)**; PDGFRβ expression expressed as intensity sum per laminin area **(B)**; and expression of the BBB tight junction proteins ZO-1 **(C)** and occludin **(D)**, expressed as intensity sum per laminin area. Representative immunomicrographs of IgG (green, scale bar = 100 µm), PDGFRβ (white, scale bar = 100 µm), and ZO-1 (aquamarine, scale bar = 10 µm) **(E)**. Data are presented as mean ± SEM. Statistical significance was determined using one-way ANOVA followed by Fisher’s LSD *post hoc* test for PDGFRβ, ZO-1, and occludin, and the Kruskal–Wallis test followed by uncorrected Dunn’s multiple-comparison test for IgG. p < 0.05; * indicates significance in the hippocampus and † indicates significance in the cortex.

Diabetes markedly increased BBB permeability in both the hippocampal formation and the cortex, as indicated by elevated parenchymal IgG abundance (Figure 5A). HA1 treatment suppressed IgG extravasation and was accompanied by increased expression of the pericyte marker PDGFRβ and the tight junction protein ZO-1 in both brain regions (Figures 5A–C,E). In contrast, although APOC3 siRNA increased cortical and hippocampal ZO-1 and PDGFRβ expression, it did not prevent IgG leakage (Figures 5A–C,E).

No significant changes were observed in occludin expression in any treatment group (Figure 5D).

### 3.6 HA1 restores astrocyte levels and exhibits antioxidative and anti-inflammatory effects

**FIGURE 6:**
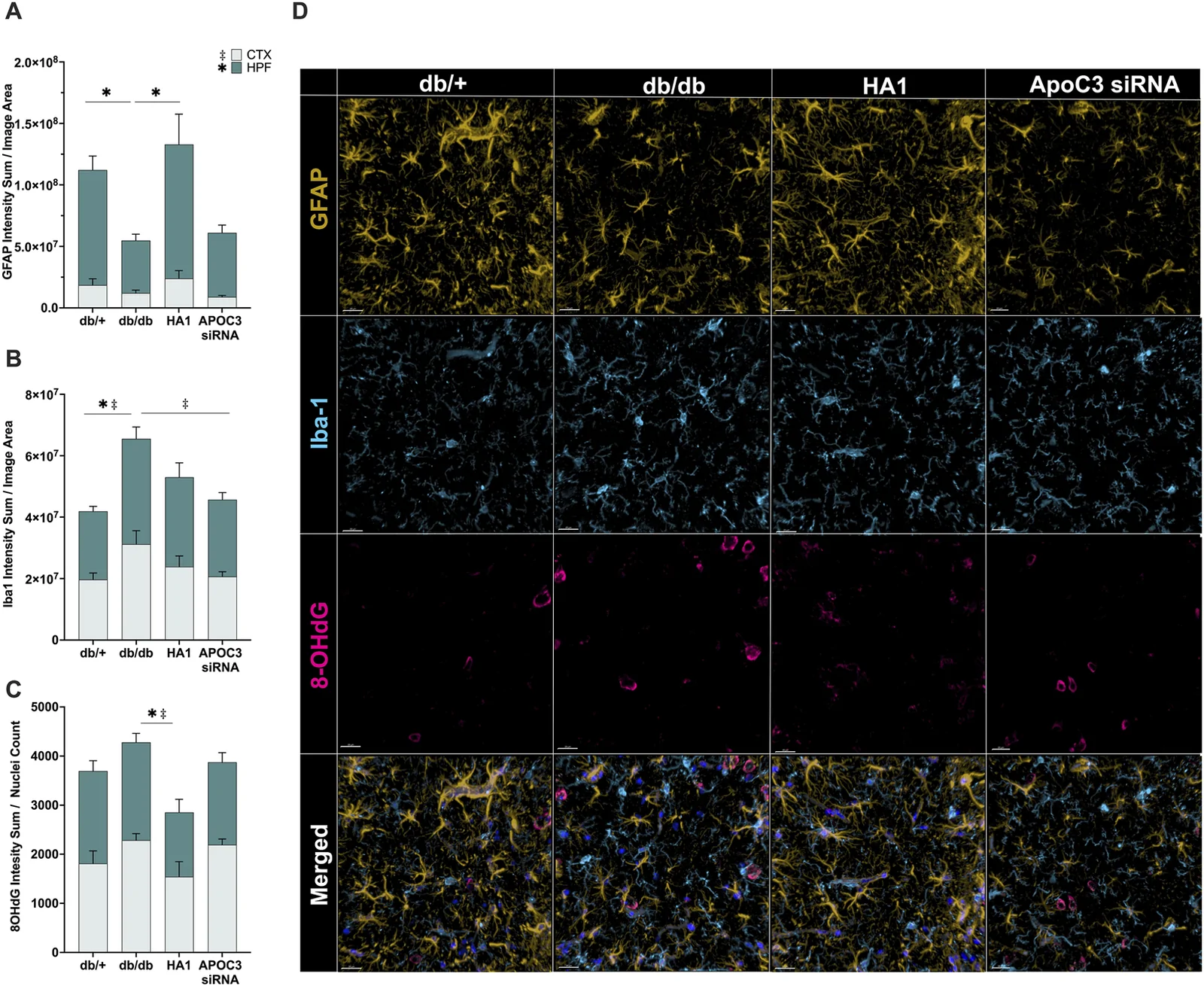
Assessment of neurovascular inflammation and oxidative stress. Mean fluorescence intensity of GFAP in the cortex and hippocampus **(A)**, Iba-1 in the cortex and hippocampus **(B)**, and 8OHdg in the cortex and hippocampus **(C)** and representative immunomicrographs of hippocampal GFAP (yellow), Iba-1 (blue), and 8-OHdG (pink) are shown **(D)**. Gamma settings were adjusted to enhance visualisation (GFAP 2.0, Iba-1 1.5). Data are presented as mean ± SEM. Scale bars represent 20 µm. Statistical significance was determined using one-way ANOVA followed by Fisher’s LSD *post hoc* test (n = 10–12). P < 0.05; * indicates significance in the hippocampus and † indicates significance in the cortex.

Diabetic db/db control mice exhibited a significant reduction in hippocampal GFAP immunoreactivity compared with db/+ controls (Figure 6A), which was normalised by HA1 treatment. In contrast, APOC3 siRNA had no significant effect on GFAP abundance (Figures 6A,D).

Increased neuroinflammation in db/db mice was indicated by elevated Iba-1 immunoreactivity, which was observed in both the cortex and hippocampus. HA1 treatment produced a minor but non-significant reduction in Iba-1 abundance (Figures 6B,D), whereas APOC3 siRNA significantly attenuated the increase in cortical Iba-1 expression.

Consistent with increased inflammatory burden, the oxidative stress marker 8-OHdG was elevated in db/db mice and was effectively attenuated by HA1 treatment, with a more modest reduction observed following APOC3 siRNA treatment (Figures 6C,D).

### 3.7 HA1 enhances axonal density within the dentate gyrus of the hippocampus

**FIGURE 7:**
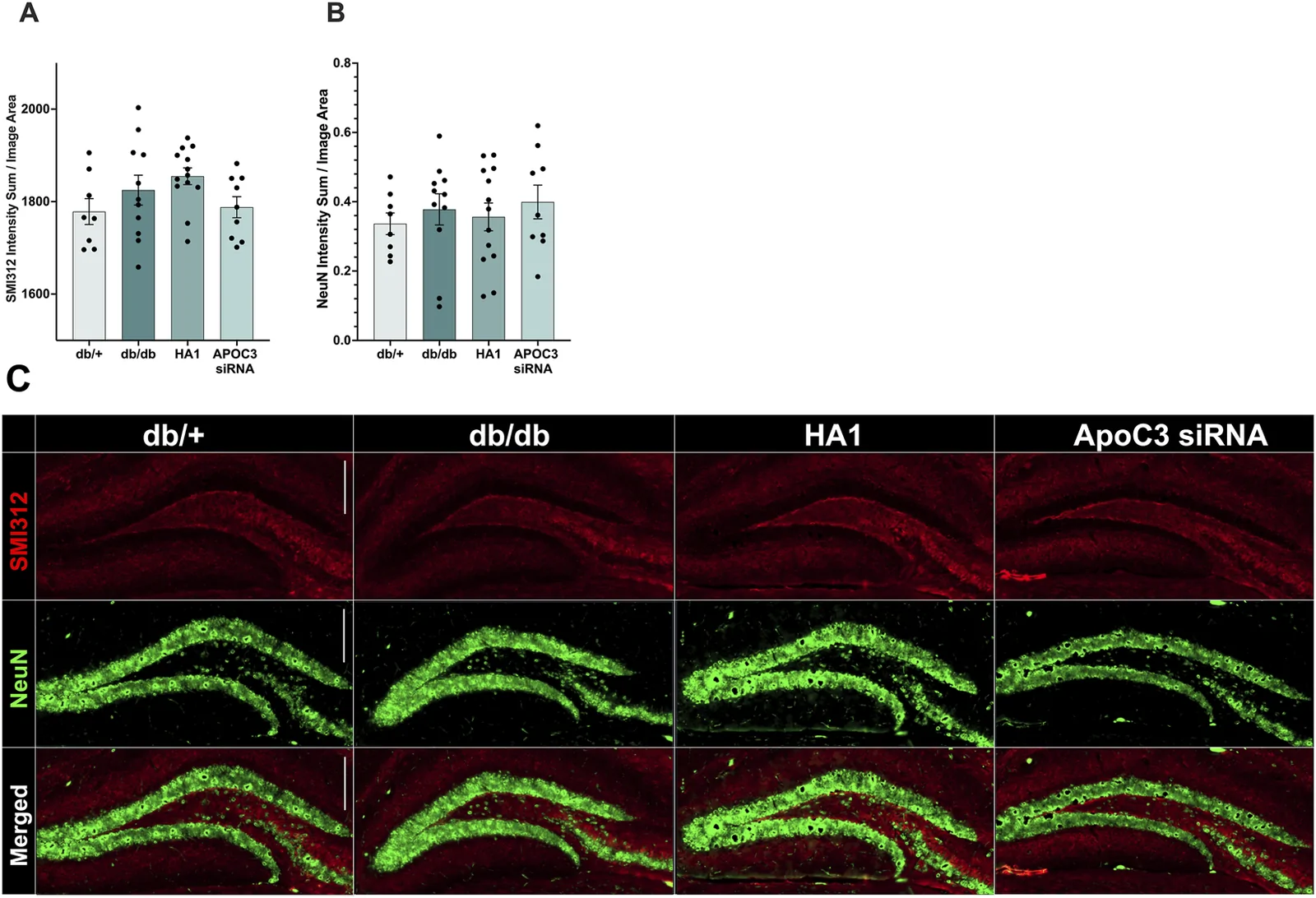
Assessment of neuronal and axonal density in the dentate gyrus of the hippocampus. Axonal density assessed by SMI312 immunoreactivity **(A)** and neuronal density assessed by NeuN immunoreactivity **(B)**, expressed as fluorescence intensity sum per image area with representative immunomicrographs of hippocampal SMI312 (red) and NeuN (green) **(C)**. Data are presented as mean ± SEM. Scale bar = 200 µm. Statistical significance was determined using one-way ANOVA followed by Fisher’s LSD *post hoc* test. (*p < 0.05, **p < 0.01, ***p < 0.001).

To determine whether improvements in neurovascular integrity were associated with changes in neuronal or axonal structure, axonal density and neuronal number were assessed in the dentate gyrus of the hippocampus using SMI312 and NeuN immunoreactivity, respectively. Diabetic db/db mice exhibited no significant differences in axonal or neuronal density compared with db/+ controls (Figures 7A–C).

Treatment with HA1 significantly increased axonal density without affecting the abundance of NeuN-positive cells. No significant changes in either axonal or neuronal markers were observed in response to APOC3 siRNA treatment.

### 3.8 HA1 and APOC3 siRNA improved anxiety-like behaviour

**FIGURE 8:**
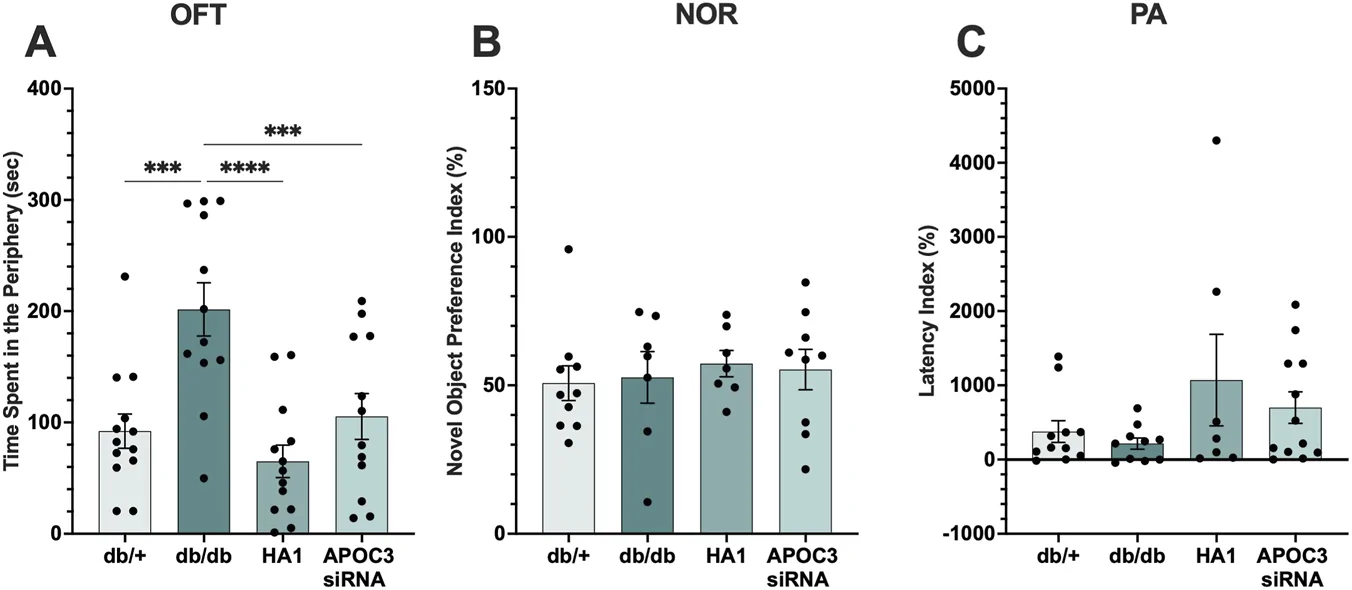
Assessment of anxiety-like behaviour and cognitive function. Anxiety-like behaviour assessed by the OFT **(A)**, short-term memory assessed by the NOR test **(B)**, and long-term memory assessed by the PA test **(C)**. Data are presented as mean ± SEM. Statistical significance was determined using one-way ANOVA followed by Fisher’s LSD *post hoc* test for OFT and NOR data, and the Kruskal–Wallis test followed by uncorrected Dunn’s multiple-comparison test for PA data. *p < 0.05, **p < 0.01, ***p < 0.001.

The risk of cognitive decline is markedly increased in T2D and is often preceded by heightened anxiety and behavioural disturbances (Pentkowski et al., 2021). At 28 weeks of age, diabetic db/db mice exhibited increased anxiety-like behaviour, as assessed by the open field test (OFT) (Figure 8A). In contrast, no impairments were detected in short-term memory, assessed by the novel object recognition (NOR) test, or in long-term memory, assessed by the passive avoidance (PA) test (Figures 8B,C).

Despite the absence of detectable cognitive deficits at this time point, treatment with either HA1 or APOC3 siRNA significantly reduced anxiety-like behaviour in the OFT (Figure 8A).

### 3.9 Plasma levels of Aβ correlate with changes in the neurovascular unit and anxiety-like behaviour

**FIGURE 9:**
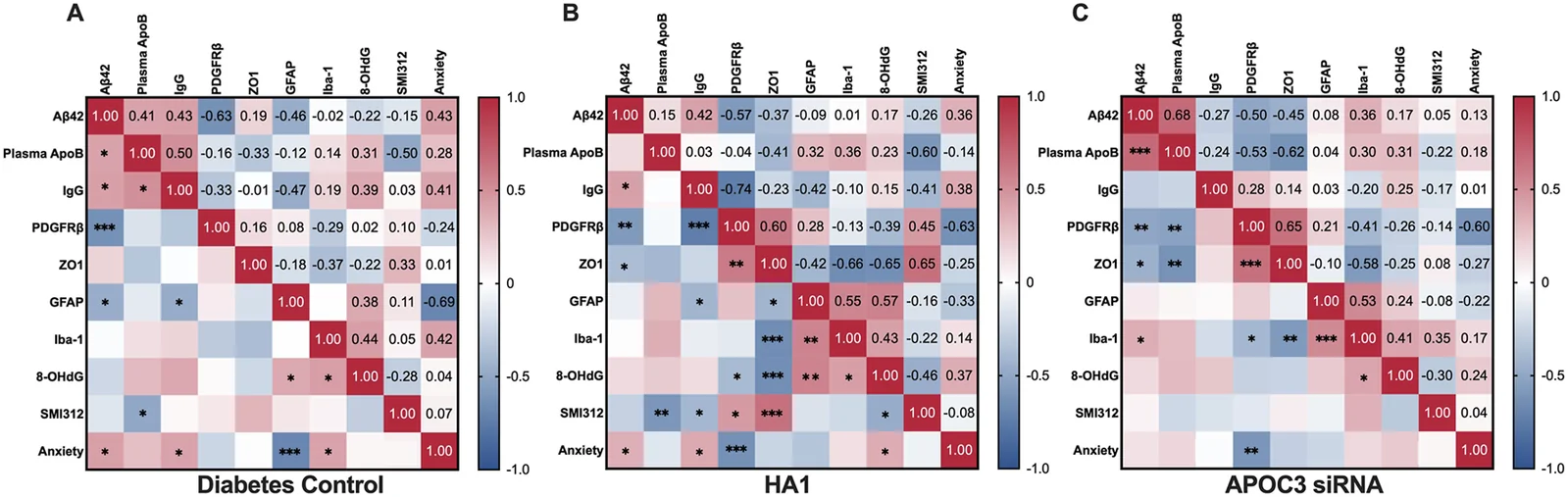
Correlation analysis between plasma Aβ_42_ and ApoB versus markers of BBB integrity. Spearman’s rank correlation analyses examining relationships between plasma Aβ_42_ and plasma ApoB with markers of BBB integrity and neurovascular function, including IgG leakage, GFAP, PDGFRβ, ZO-1, SMI312, and anxiety-like behaviour. Analyses were performed using data from combined db/+ and untreated db/db mice **(A)**, db/db mice treated with HA1 **(B)**, and db/db mice treated with APOC3 siRNA **(C)**. Spearman’s correlation coefficients (r) with 95% confidence intervals are shown in the upper triangle of each matrix, and corresponding statistical significance values are shown in the lower triangle (*p < 0.05, **p < 0.01, ***p < 0.001; n = 19–26).

Spearman’s correlation analysis, shown in Figure 9, examined the associations between plasma ApoB and plasma Aβ_42_ levels and markers of the neurovascular unit, neuroinflammation, oxidative stress, axonal density, and anxiety-like behaviour.

In control mice, positive correlations were observed between plasma ApoB and Aβ_42_ levels and both BBB permeability, assessed by IgG leakage, and anxiety-like behaviour (Figure 9A). Plasma Aβ_42_ levels and BBB leakage were inversely correlated with astrocyte abundance, assessed by GFAP immunoreactivity, and pericyte abundance, assessed by PDGFRβ immunoreactivity, while BBB leakage was positively associated with 8-OHdG, a marker of oxidative stress. Anxiety-like behaviour was also positively correlated with BBB leakage, and increased microglial number, assessed by Iba-1 immunoreactivity, and inversely correlated with astrocyte abundance. These findings indicate that elevated circulating lipoprotein and Aβ_42_ levels are associated with BBB leakage, glial dysregulation, oxidative stress, and anxiety-like behaviour.

In HA1-treated mice, correlation analyses indicated associations among plasma Aβ_42_ levels, BBB integrity, axonal density, and anxiety-like behaviour (Figure 9B). Specifically, plasma Aβ_42_ levels showed robust positive correlations with parenchymal IgG leakage and anxiety-like behaviour, and negative correlations with pericyte abundance and ZO-1 expression. Increased BBB permeability was also associated with reduced pericyte, astrocyte, and axonal density, as well as increased anxiety-like behaviour. Anxiety also showed a positive association with oxidative stress markers. These findings indicate that the lower plasma Aβ_42_ levels observed with HA1 treatment were accompanied by a more favourable neurovascular and behavioural profile.

In APOC3 siRNA-treated mice (Figure 9C), plasma Aβ_42_ and ApoB levels did not correlate with IgG leakage but remained associated with ZO-1 expression and pericyte abundance. Pericyte abundance was also inversely associated with anxiety-like behaviour. These findings suggest that APOC3 siRNA was associated with selective changes in vascular support markers and behavioural outcomes, without a corresponding reduction in BBB permeability.

## 4 Discussion

This study showed a significant increase in the intestinal abundance of both ApoB and Aβ in db/db mice, consistent with altered lipoprotein and Aβ metabolism in diabetes. These changes are likely due to synergistic effects of insulin resistance, diabetic hyperphagia and intestinal hypertrophy. Meanwhile, plasma analyses revealed increases in total plasma ApoB, Aβ_42_, and Aβ oligomers, while Aβ_40_ levels remained unchanged. The elevated plasma abundance of lipoprotein and Aβ_42_ may reflect increased intestinal lipoprotein and Aβ production, coupled with delayed clearance via high-affinity receptor pathways (Martins et al., 2002), thereby favouring a greater propensity for Aβ oligomer formation. Taken together, this study, alongside previous research (Wiest et al., 2021; Meakin et al., 2020; Rolandsson et al., 2024; Sharif et al., 2025), supports the hypothesis that T2D is associated with impaired peripheral metabolism of lipoprotein and Aβ, leading to chronically exaggerated vascular exposure to circulating postprandial Aβ.

Research in AD increasingly links BBB disruption to increased vascular exposure to elevated levels of circulating lipoprotein-Aβ (Lam et al., 2021; Takechi et al., 2008; D’Alonzo et al., 2023). Consistent with this, the diabetic db/db mice exhibited significantly compromised BBB integrity, as evidenced by increased parenchymal extravasation of IgG. However, the observed increase in capillary permeability was not accompanied by measurable reductions in the expression levels of the tight junction proteins ZO-1 and occludin. This suggests that increased permeability may reflect functional changes not captured by total tight-junction abundance, including altered junctional localisation (Rahman et al., 2018), post-translational modification (Cummins, 2012), impaired cytoskeletal anchoring (Tietz and Engelhardt, 2015) or disrupted endothelial signalling (Stamatovic et al., 2016). Furthermore, in early diabetes, tight junction protein expression has been shown to initially increase as a compensatory response to maintain barrier integrity (Xu et al., 2024) but this response may ultimately fail to sustain proper BBB function due to abnormal localisation (Rom et al., 2020). Moreover, BBB integrity is dependent on the structural and functional support provided by pericytes and astrocytes and is particularly vulnerable to oxidative stress and inflammation. In this study, db/db mice exhibited reduced pericyte coverage of cerebral vessels and decreased astrocyte abundance, along with elevated markers of reactive microgliosis.

In this study, the evolution of BBB dysfunction in db/db mice had not progressed to overt neurodegeneration or loss of axonal density. Cognitive performance also remained unaffected, with no deficits observed in either long-term or short-term memory, as assessed by the PA and NOR tests. These findings align with our previous studies, which indicate that BBB impairment precedes the onset of overt neurodegenerative and cognitive symptoms (Takechi et al., 2017). However, behavioural analysis using the OFT revealed a significant increase in anxiety-like behaviour in db/db mice. The development of anxiety- and depressive-like phenotypes is considered to represent prodromal symptoms of AD (Pentkowski et al., 2021), and may be driven by increased permeation of proinflammatory cytokines into the brain parenchyma and activation of cerebral glial cells (Welcome, 2020).

Consistent with this, Spearman correlation analysis indicated significant associations between anxiety-like behaviours and increased capillary permeability, microglial activation, and astrocyte loss. Additionally, elevated plasma levels of lipoprotein and Aβ_42_ were associated with BBB dysfunction, degeneration of astrocytes and pericytes, as well as increased anxiety-like behaviour. These associations raise the possibility that elevated circulating Aβ_42_, in the context of impaired lipoprotein metabolism, is linked to BBB dysfunction and behavioural deficits through neuroinflammatory mechanisms and altered astrocyte and pericyte homeostasis. Indeed, previous studies have demonstrated that hippocampal astrocytes can exert anxiolytic effects by enhancing synaptic activity (Cho et al., 2022), while both astrocytes and pericytes are critical for maintaining BBB integrity and responding to pro-inflammatory stimuli, thereby modulating the neuroinflammatory environment (Bhattacharya et al., 2020).

APOC3 siRNA accelerates the metabolism and clearance of nascent TRLs by promoting endothelial lipoprotein lipase–mediated hydrolysis and conversion to triglyceride-depleted particles, which are then cleared predominantly by high-affinity hepatic pathways (Giammanco et al., 2023). Consistent with accelerated TRL clearance, APOC3 siRNA significantly reduced plasma ApoB, Aβ_42_, and Aβ oligomers. Because intestinal Aβ abundance was unchanged, these reductions are unlikely to be explained by reduced intestinal amyloid synthesis. Rather they are consistent with the hypothesis that APOC3 siRNA-mediated reductions in ApoB-containing lipoproteins may lower the circulating Aβ pool associated with these particles. This is further supported by correlation analyses demonstrating a preserved association between plasma Aβ_42_ and plasma ApoB following APOC3 siRNA treatment, despite disruption of the association between intestinal Aβ and circulating Aβ_42_.

Notably, while APOC3 siRNA improved several structural and inflammatory readouts—greater pericyte coverage, increased ZO-1 immunoreactivity, reduced microgliosis, and attenuated anxiety-like behaviour—it did not reduce capillary leakage, despite lowering steady-state plasma amyloid. This dissociation highlights the need to consider absolute quantitative measures of metabolism (i.e., concentration × turnover) rather than relying solely on steady-state plasma levels. Postprandial (chylomicron) lipid flux is orders of magnitude greater than endogenous (VLDL-derived) hepatic lipid flux (Mamo et al., 1997; Sullivan et al., 2004), which may also apply to lipoprotein-amyloid. Additionally, while APOC3 siRNA has not been shown to inhibit lipoprotein biogenesis, increases in chylomicron secretion have been reported (Syed-Abdul et al., 2023). Such effects may increase lipoprotein flux and alter circulating Aβ exposure, potentially offsetting the vascular benefits of lowered steady-state plasma amyloid levels.

Another potential factor that may influence the vascular effects of APOC3 siRNA is heightened microvascular exposure to non-esterified fatty acids and other lipolysis products, which are liberated in greater amounts with APOC3 siRNA treatment due to increased triglyceride hydrolysis. Consistent with this possibility, previous studies have shown that lipolysis products from TRLs can damage endothelial barrier function, induce apoptosis in endothelial cells, and elevate BBB permeability in murine models (Ng et al., 2016; Eiselein et al., 2007).

HA1, however, preserved neurovascular integrity and mitigated behavioural deficits in db/db diabetic mice. Specifically, it increased tight junction protein expression, preserved astrocyte and pericyte abundance and prevented cerebral oxidative stress and microglial activation. These effects were accompanied by a significant reduction in BBB leakage and anxiety-like behaviour, as well as increased axonal density. In addition, HA1 markedly reduced intestinal Aβ abundance and plasma levels of the toxic Aβ_42_ and Aβ oligomers without altering plasma lipoprotein concentrations. Collectively, these findings align with studies indicating that exposure to lipoprotein-Aβ, rather than the lipoprotein itself, regulates capillary function (Takechi et al., 2008; Qahwash et al., 2007). For example, Meakin et al. (Meakin et al., 2020) demonstrated that elevated plasma Aβ_42_ levels in high-fat-fed, obese, and hyperglycaemic mice induced vascular dysfunction comparable to that observed in chow-fed mice following intravenous administration of physiological doses of Aβ_42_.

Correlation analyses further revealed that reductions in plasma Aβ_42_ in HA1-treated mice were associated with improved BBB integrity, increased ZO-1 and pericyte levels and reduced anxiety-like behaviour. In addition, higher astrocyte and pericyte levels correlated with enhanced BBB integrity and axonal density and reduced anxiety. These findings suggest that HA1 may help preserve BBB function and mitigate behavioural deficits, in part by reducing circulating Aβ and thereby decreasing the pool available for association with circulating lipoproteins, while also maintaining neurovascular and glial components.

However, this study was not powered to elucidate how HA1 modulates the synthesis and secretion of Aβ, which is secreted as an apoprotein of nascent TRLs (Koudinov and Koudinova, 1997). It remains unclear whether, in enterocytes and hepatocytes, Aβ is derived from precursor proteins found in intracellular and plasma membranes. Colocalisation studies of intestinal Aβ with ApoB suggest that Aβ may not originate from the plasma membrane in enterocytes (Galloway et al., 2009).

Previous studies in high-fat-fed models have demonstrated that native probucol attenuates diet-induced chylomicron-Aβ secretion, accompanied by preservation of BBB integrity and cognitive function (Sharif et al., 2024; Takechi et al., 2013; Takechi et al., 2014; Santos et al., 2012). A significant consideration is that HA1 is a novel chemical entity conjugated to probucol at the C24 position of the LCA moiety at the phenolic end of probucol. As a result, HA1 may exert effects beyond modulation of Aβ alone. LCA itself may mediate biological effects that synergise with probucol’s pleiotropic properties. It can activate the FXR and TGR5 signalling pathways, which induce the release of glucagon-like peptide-1 (GLP-1) (Chiang and Ferrell, 2020). GLP-1 has been shown to reduce food intake, promote weight loss, increase insulin secretion, and suppress lipid synthesis (Hag et al., 2007). Moreover, bile acid-mediated FXR activation has been shown to lower postprandial lipemia (Farr et al., 2020; Stankovic, 2019) and downregulate microsomal triglyceride transfer protein expression (Hirokane et al., 2004; Kalaany and Mangelsdorf, 2006). Consistent with these reported actions, HA1 treatment in the present study was associated with reduced weight gain, lower plasma glucose and triglyceride levels, and increased insulin concentrations. Beyond FXR and TGR5 signalling, LCA is also a potent activator of the vitamin D receptor, which may further contribute to the multifactorial metabolic and vascular effects observed with HA1 treatment (Belorusova et al., 2014).

More broadly, this study underscores the importance of postprandial lipoprotein metabolism in modulating peripheral Aβ changes in diabetes. After the ingestion of fats, postprandial chylomicrons may influence the circulating lipoprotein-amyloid environment and the transport and deposition of amyloid peptides (Takechi et al., 2008). In individuals with diabetes, postprandial aberrations in lipoprotein metabolism often also result in increased production and impaired clearance of VLDL, potentially exacerbating amyloid accumulation in the brain (Hirano, 2018; van Arendonk et al., 2023).

While the findings of this study provide important insight into the relationships between lipoprotein and Aβ metabolism, neurovascular function, and behavioural outcomes in diabetes, several limitations should be considered. First, although circulating Aβ and ApoB were measured, TRL- Aβ was not directly isolated. Second, associations between circulating Aβ/ApoB measures, BBB integrity, neurovascular markers, and behaviour require mechanistic validation. Third, only male mice were studied, and unrestricted access to food before sample collection may have influenced circulating lipoprotein- and Aβ-related measures through variation in feeding state and postprandial metabolism. Finally, future studies incorporating TRL isolation, kinetic and turnover analyses, controlled feeding conditions, and both male and female mice would help define particle-specific Aβ handling, treatment-related mechanisms, and sex-dependent responses.

## 5 Conclusion

The findings of this study reinforce the hypothesis that chronic exposure to Aβ in the context of altered lipoprotein metabolism contributes to neurovascular dysfunction in T2D. Of the therapeutic agents tested, the second-generation probucol analogue HA1 demonstrated superior efficacy in improving metabolic profiles and reducing plasma levels of Aβ_42_ and Aβ oligomers. These changes were accompanied by preserved BBB integrity and astrocyte and pericyte homeostasis and reduced oxidative stress and microglial activation, and attenuated anxiety-like behaviours. In contrast, while APOC3 siRNA showed partial effects on metabolic parameters and amyloid clearance, it was less effective in preserving neurovascular integrity. The multifactorial benefits of HA1, including its potential synergy with bile acid signalling pathways, suggest broader therapeutic applicability beyond diabetes-induced neurovascular pathology, offering new avenues for the treatment of neurodegenerative diseases characterised by amyloidosis and vascular dysfunction. Further investigation into the precise mechanisms by which HA1 modulates amyloid metabolism will be critical for advancing its therapeutic development.

## Data Availability

The raw data supporting the conclusions of this article will be made available by the authors, without undue reservation.
